# Protocol for a double-blind placebo-controlled randomised controlled trial assessing the impact of oral semaglutide in amyloid positivity (ISAP) in community dwelling UK adults

**DOI:** 10.1136/bmjopen-2023-081401

**Published:** 2024-06-20

**Authors:** Ivan Koychev, Amanda I Adler, Paul Edison, Brian Tom, Joanne E Milton, Joe Butchart, Adam Hampshire, Charles Marshall, Elizabeth Coulthard, Henrik Zetterberg, Peter Hellyer, Francesca Cormack, Benjamin R Underwood, Catherine J Mummery, Rury R Holman

**Affiliations:** 1 Department of Psychiatry, University of Oxford, Oxford, UK; 2 Diabetes Trials Unit, Radcliffe Department of Medicine, University of Oxford, Oxford, UK; 3 Faculty of Medicine, Department of Brain Sciences, Imperial College London, London, UK; 4 Medical Research Council Biostatistics Unit, University of Cambridge, UK; 5 Royal Devon University Healthcare Foundation Trust, Exeter, UK; 6 University of Exeter Medical School, Exeter, UK; 7 Preventive Neurology Unit, Wolfson Institute of Population Health, Queen Mary University of London, London, UK; 8 Bristol Medical School, University of Bristol, Bristol, UK; 9 Department of Psychiatry and Neurochemistry, Institute of Neuroscience and Physiology, the Sahlgrenska Academy at the University of Gothenburg, Mölndal, Sweden; 10 Clinical Neurochemistry Laboratory, Sahlgrenska University Hospital, Mölndal, Sweden; 11 Department of Neurodegenerative Disease, UCL Institute of Neurology, Queen Square, London, UK; 12 UK Dementia Research Institute at UCL, London, UK; 13 Hong Kong Center for Neurodegenerative Diseases, Clear Water Bay, Hong Kong, People's Republic of China; 14 Wisconsin Alzheimer’s Disease Research Center, University of Wisconsin School of Medicine and Public Health, University of Wisconsin-Madison, Madison, WI, USA18 Dementia Research Centre, Institute of Neurology, University College London, Queen Square, London, UK; 15 Cambridge Cognition Ltd, Cambridge, UK; 16 Department of Psychiatry, University of Cambridge, Cambridge, UK; 17 Cambridgeshire and Peterborough NHS Foundation trust, Cambridge, UK; 18 Dementia Research Centre, Institute of Neurology, University College London, Queen Square, London, UK

**Keywords:** dementia, nuclear medicine, protocols & guidelines

## Abstract

**Introduction:**

Glucagon-like peptide-1 receptor agonists (GLP-1 RAs), currently marketed for type 2 diabetes and obesity, may offer novel mechanisms to delay or prevent neurotoxicity associated with Alzheimer’s disease (AD). The impact of semaglutide in amyloid positivity (ISAP) trial is investigating whether the GLP-1 RA semaglutide reduces accumulation in the brain of cortical tau protein and neuroinflammation in individuals with preclinical/prodromal AD.

**Methods and analysis:**

ISAP is an investigator-led, randomised, double-blind, superiority trial of oral semaglutide compared with placebo. Up to 88 individuals aged ≥55 years with brain amyloid positivity as assessed by positron emission tomography (PET) or cerebrospinal fluid, and no or mild cognitive impairment, will be randomised. People with the low-affinity binding variant of the rs6971 allele of the Translocator Protein 18 kDa (TSPO) gene, which can interfere with interpreting TSPO PET scans (a measure of neuroinflammation), will be excluded.

At baseline, participants undergo tau, TSPO PET and MRI scanning, and provide data on physical activity and cognition. Eligible individuals are randomised in a 1:1 ratio to once-daily oral semaglutide or placebo, starting at 3 mg and up-titrating to 14 mg over 8 weeks. They will attend safety visits and provide blood samples to measure AD biomarkers at weeks 4, 8, 26 and 39. All cognitive assessments are repeated at week 26. The last study visit will be at week 52, when all baseline measurements will be repeated. The primary end point is the 1-year change in tau PET signal.

**Ethics and dissemination:**

The study was approved by the West Midlands—Edgbaston Research Ethics Committee (22/WM/0013). The results of the study will be disseminated through scientific presentations and peer-reviewed publications.

**Trial registration number:**

ISRCTN71283871.

**STRENGTHS AND LIMITATIONS OF THIS STUDY**

- Interventional study in a predementia population with pathological evidence of potential Alzheimer’s disease (AD).
- Randomisation in a trial setting minimises potential between-group difference at baseline.
- Measures of brain tau will test the primary hypothesis that glucagon-like peptide-1 receptor agonists interact with core AD pathophysiology.
- The study is not powered to establish the efficacy of semaglutide in preclinical AD.

## Introduction

 Alzheimer’s disease (AD), characterised by synaptic dysfunction and neurodegeneration, is thought to be triggered by the accumulation in the brain of amyloid plaques and neurofibrillary tangles, aggregates of hyperphosphorylated τ proteins.^1^ This process likely begins decades before symptoms of AD become evident, with supra-threshold levels of cortical amyloid triggering the AD pathophysiological cascade.^1^ ‘Amyloid positivity’ on positron emission tomography (PET) scans or in cerebrospinal fluid (CSF) assays in cognitively healthy individuals is diagnostic of the preclinical stages of AD.^2^ While treatments designed to reduce accumulations of these abnormal levels of protein have recently been shown to associate with modest clinical benefit in patients with AD (mild cognitive impairment (MCI) or dementia), they are nonetheless associated with significant adverse effects, and delivering them in routine clinical practice requires a sizeable investment and reorganisation of services. Therefore, secondary prevention strategies in people with preclinical disease is unlikely to rest primarily on amyloid clearance approaches. An alternative strategy for both treatment and secondary AD prevention is the repurposing of existing compounds which has shown efficacy elsewhere (eg, COVID-19).^3^ ^4^ In relation to AD, this strategy is underdeveloped.

Repurposing compounds reduces risk, cost and time to providing a new treatment.^4^ Drug development risk is reduced through a well-established safety profile and thus bypasses the need for early preclinical safety testing and iterative chemical compound optimisation. Additionally, significant knowledge of clinical safety can be gained from existing literature, pharmacovigilance, and clinical experience. Further advantages of this strategy include real-world evidence of effectiveness, and biosamples from previous trials. For AD, repurposing is particularly relevant as pathways thought to propagate both AD and non-AD neurodegeneration (neuroinflammation, central nervous system insulin resistance (IR) and cerebrovascular pathology) can already be targeted by approved compounds and thus represent viable treatment targets.

Several compounds have been identified as high priority targets for repurposing in AD. Among these, a class of medications developed for the treatment of type 2 diabetes (T2D) known as glucagon-like peptide-1 receptor agonists (GLP-1 RAs) offers promise through strong epidemiological and preclinical data suggesting neuroprotective effects. GLP-1 RAs are incretins which enhance glucose-dependent insulin secretion, slow gastric emptying and reduce both postprandial glucagon secretion and food intake.^5^ The effect is reduced postprandial blood glucose without the risk of hypoglycaemia. They are currently marketed for glycaemic control in T2D, and for weight loss in individuals with obesity or overweight individuals with comorbidities).^6^ Importantly for dementia research, these classes of compounds cross the blood-brain barrier (BBB) and have been shown to be neuroprotective in animal models of neurodegeneration^7 8^; additionally, they can be given safely in non-diabetic populations due to the low risk of hypoglycaemia.

The evidence for potential GLP-1 RA efficacy in AD comes from pharmacoepidemiological studies that have demonstrated an association of their use in T2D and reduced incidence of dementia.^9 10^ A nested case-control study based on dementia diagnosis within a cohort of 176 people with T2D showed that GLP-1 RAs were associated with a significantly reduced odds of dementia after adjusting for demographic confounders, acute and chronic diabetes complications and use of other types of antidiabetic agents (OR 0.58, 95% CI 0.42 to 0.81, relative to placebo).^10^ In addition, increasing exposure to GLP-1 RAs over time resulted in further gradual decrease in the odds of developing dementia. A paper reporting the dementia-related outcomes in 15 820 people with T2D from three double-blind randomised placebo-controlled cardiovascular trials (Liraglutide Effect and Action in Diabetes: Evaluation of Cardiovascular Outcome Results (LEADER), Peptide Innovation for Early Diabetes Treatment 6 (PIONEER 6) and Trial to Evaluate Cardiovascular and Other Long-term Outcomes with Semaglutide in Subjects with Type 2 Diabetes (SUSTAIN-6)) and a Danish healthcare register-based cohort (120 054 individuals) found similar results.^9^ The authors reported a relative risk reduction for dementia with HRs for GLP-1 RAs of 0.47 (95% CI 0.25 to 0.86) and 0.89 (95% CI 0.86 to 0.93) relative to other diabetes treatments and placebo in the trials and cohort data, respectively. Increase in yearly exposure to GLP-1 RAs was associated with further benefit related to dementia that primarily affected individuals ≤70 years of age.

Mechanisms for the potential disease-modifying action of GLP-1 RAs regarding dementia is probably multifactorial. A pilot trial in patients with AD showed liraglutide, compared with placebo, decreased the rate of decline of brain glucose metabolism^11^ and increased the capacity of the BBB to transfer glucose.^12^ While the pilot trial did not find an effect on amyloid accumulation in a secondary analysis, there is now evidence that tau protein changes are more tightly related to IR.^13^ A preclinical study demonstrated that loss of tau function (tau deletion in knockout mice) was associated with impaired hippocampal and hypothalamic responses to insulin.^14^ This builds on evidence demonstrating that tau hyperphosphorylation leads to neuronal IR and intracellular insulin accumulation.^15^ These results add to the growing appreciation that tau’s physiological role is significantly broader than neuronal structure support and includes regulation of insulin signalling, in addition to DNA protection from oxidative stress^16^ and control of neuronal excitability.^17^ Alternative potential mechanisms underlying GLP-1 RAs’ effects in dementia are through inflammatory and cerebrovascular factors; neuroinflammation is a key driver of AD pathology^18^ with GLP-1 RAs having been shown to regulate both systemic^19^ and neuroinflammation.^20^ For example, in a post hoc analysis of three trials of semaglutide, the agent was shown to reduce C reactive protein by 39%–48% after 68 weeks of treatment. In addition, cardiovascular risk factors mediate the risk of AD and dementia in general.^21^ GLP-1 RAs have consistently been shown to reduce the risk of major cardiovascular and cerebrovascular events, which may in turn translate into lower risk of dementia.^22 23^ Specifically, in the EXSCEL trial, exenatide reduced the occurrence of death caused by a composite of vascular events (cardiovascular causes, non-fatal myocardial infarction or non-fatal stroke),^22^ while SUSTAIN 6 and PIONEER 6 trials found that semaglutide reduced the incidence of major cardiovascular events in patients with T2D.^23^ Thus, there remains a need for research into the mechanism through which GLP-1 RAs may be beneficial in dementia. The knowledge gained could guide the design of trials aimed at demonstrating GLP-1 RA efficacy, particularly where a specific subgroup (eg, individuals with high cardiovascular risk or high levels of systemic inflammation or genetic risk for AD) is shown to be most likely to benefit from them.

### Methods and analysis

ISAP is a randomised, double-blind, placebo-controlled parallel-group, superiority trial of oral semaglutide given over a period of 12 months that aims to clarify the mechanism through which GLP-1 RAs may impact AD pathophysiology. It is recruiting adults in preclinical stages of AD through having high levels of cortical amyloid as assessed by PET. The study start date was 1 March 2021 with a planned end date of 15 March 2026.

#### Primary objective

The primary objective is to evaluate the impact of oral semaglutide compared with placebo on tau accumulation in amyloid-positive dementia-free ageing adults.

#### Primary end point

The primary end point for the study is cortical tau accumulation over 1 year as assessed by tau PET.

#### Secondary analyses

We will examine the effects of semaglutide compared with placebo on brain neuroinflammation as determined by the Translocator Protein 18 kDa (TSPO) PET and plasma assays of biomarkers relevant to dementia, cognition, neurodegeneration as determined by MRI, AD plasma biomarkers and physical activity variation as determined by wrist-worn actigraphy.

#### Exploratory analyses

Exploratory analyses of plasma samples using multidimensional protein assays will include established AD biomarkers, such as amyloid β 42 over 40 ratio (Aβ42/Aβ40), phosphorylated tau forms (181 and 217), neurofilament light as a neurodegeneration marker and glial fibrillar acidic protein (GFAP) as an astrocytic activation marker.^24^ We will also investigate the plasma of individuals who at screening have amyloid-negative PET scans and are excluded from randomisation to (1) inform screening procedures for future predementia AD trials and (2) define how individuals who are not eligible for the trial differ from those included to help inform the generalisability of the study results. Plasma will be retained for further proteomic exploratory analyses. See table 1 for a summary of outcome measures.

**Table 1:** Primary, secondary and exploratory outcomes

Outcomes	Method	Measurements
Primary		
Annualised cortical tau change	PET tau	Baseline, week 52
Secondary and exploratory outcome measures		
Cortical neuroinflammatory signal	TSPO PET	Baseline, week 52
Plasma biomarkers of neuroinflammation	Plasma GFAP	Screening, baseline, weeks 4, 8, 26, 39 and 52
Plasma AD biomarkers	Plasma p-tau181 and Aβ42/40	Screening, baseline, weeks 4, 8, 26, 39 and 52
Cognition (in-clinic)	ACE-III, Cambridge Cognition CANTAB battery	Baseline, weeks 26 and 52
Cognition (remote)	Cognitron	Baseline, weeks 26 and 52
Adverse events	Presence of adverse events	Baseline, weeks 4, 8, 26, 39, 52 and follow-up call
Neurodegeneration (imaging)	Hippocampal MRI volume	Baseline and 52 weeks
Neurodegeneration (plasma)	Plasma NFL	Screening, baseline, weeks 4, 8, 26, 39 and 52
Depression and anxiety levels	CES-D and HAI scales	Baseline, week 52
Distress at AD risk disclosure	Genetic testing for AD scale	Weeks 26 and 52
Quality of life	EQ-5D-5L scale	Baseline, week 52
Level and pattern of physical activity, and circadian rhythms	Wrist-worn actigraphy	Baseline, week 52

ACE-IIIAddenbrooke’s Cognitive AssessmentADAlzheimer’s diseaseCES-DCenter for Epidemiological Studies DepressionGFAPglial fibrillar acidic proteinHAIHealth Anxiety InventoryNFLneurofilament lightPETpositron emission tomographyTSPOTranslocator Protein 18 kDa

#### Study subjects

Amyloid-positive community dwelling UK-based volunteers (determined through PET amyloid scanning or CSF) of both sexes aged ≥55 years with no or MCI (evidenced through a score of 0.5 or below on the Clinical Dementia Rating (CDR) scale). See online supplemental appendix 1 for a full list of trial inclusion and exclusion criteria.

We will recruit using existing recruitment resources (eg, electronic research registers or research volunteer lists) or electronic healthcare records in primary or secondary care (subject to a valid consent to be approached for research). The stratification algorithm we use combines diagnosis of MCI, carriage of the apolipoprotein E (*APOE*) ε4 allele, family history of dementia in a first-degree relative, advancing age (treated as continuous variable), sex and where available cognitive function and AD biomarkers in plasma.

### Patient and public involvement

Members of the public were involved in the design stages of the study during a patient and public involvement (PPI) group meeting on 12 March 2020. The PPI input informed the study design and prioritising amyloid PET over lumbar puncture as a screening method. A member of the public also sits on the ISAP trial steering committee.

## Study procedures

### Screening visit

At the screening visit, subjects provide written informed consent with a medically trained study investigator (consent form available as a online supplemental file) and undergo a medical history and physical examination, interview for the presence of dementia (CDR scale) and blood samples. These samples inform eligibility, determine TSPO allele carriership (low-affinity binding variant of the rs6971 allele of the TSPO gene is an exclusion criterion) and provide samples for research biomarker analyses. An informant indicated by the participant is approached to collect information relevant to the CDR scale. Individuals that meet study criteria then undergo amyloid PET testing to determine their amyloid status.

For each PET scan, participants receive an intravenous injection of the appropriate radiotracer (florbetaben or if unavailable, florbetapir or flutemetamol with a maximum of 300 MBq activity per scan) with data acquisition in the region of 20–30 min (depending on tracers) following an uptake period appropriate for the particular radioligand and its kinetics.Amyloid scans are read clinically and full quantification is also performed.

Amyloid status will also be possible to establish alternatively through CSF sampling (subject to ethical amendment currently under consideration) depending on study logistics and participant preference. A minimum of 500 μL sample will be analysed using the Lumipulse G600II automated assay (Fujirebio) for Aβ42 and Aβ40. Aβ42/Aβ40 ratio will be used due to previously demonstrated high concordance with amyloid PET relative to Aβ42 alone and defined a positivity cut-off of 0.065.^25^

### Baseline visit

Participants eligible for the study based on screening procedures are invited to a baseline study visit when amyloid status (positive or negative) is disclosed to them in accordance with the ISAP study Amyloid Disclosure standard operating procedure. Baseline measures of cognition are recorded using computerised testing (CANTAB battery focusing on attention, episodic memory, processing speed, working memory and executive function) and the Addenbrooke’s Cognitive Examination III, 2017 (ACE-III). Participants complete questionnaires probing health-related quality of life (EQ-5D-5L), health anxiety (Health Anxiety Inventory), affective symptoms (Center for Epidemiological Studies Depression Scale) and disclosure of dementia risk. Participants are then allocated at random to semaglutide or matching placebo in a 1:1 ratio. Treatment assignments are performed using a computerised procedure with minimisation (adaptive stratified sampling) based on T2D (yes/no), MCI (yes/no) and trial site to maintain balance between treatment groups. Participant replacement is not permitted.

Before initiating study treatment, participants are required to undergo tau PET, TSPO PET and MRI scanning as well as completing their remote actigraphy and cognition assessments.

### Tau positron emission tomography

A target dose of 185 MBq using one of three tau PET tracers depending on availability ([18F]PI-2620; [18F]AV1451; [18F]MK-6240) is administered intravenously. Image acquisition takes place 30 min after appropriate uptake time depending on the ligand. PET images are co-registered to their T1-weighted MRI, and fully quantified.

### Translocator Protein 18 kDa positron emission tomography

A target dose of 185 MBq 18F-DPA714 is injected intravenously followed by up to 60 min duration of image acquisition. The images are co-registered to a T1-weighted MRI, and then transformed into Montreal Neurological Institute space and fully quantified.

### Magnetic resonance imaging

The MRI acquisition protocol consists of the following sequences: T1-weighted imaging, three-dimensional T2-fluid-attenuated inversion recovery (FLAIR), axial T2-weighted, diffusion weighted and T2*-weighted imaging. The analysis of the MRI protocol will comprise measures of (i) macrostructural change derived from volumetric T1-weighted imaging including global and regional atrophy rates; (ii) microstructural pathology and loss of connectivity through change in grey and white matter diffusivity (diffusion-weighted imaging derived from fractional anisotropy, axial and radial diffusivity); (iii) vascular burden and microhaemorrhages derived from FLAIR, T2-weighted and quantitative susceptibility mapping; (iv) total and regional white matter hyperintensities volumes as well as Fazekas visual scoring of cerebrovascular burden.

### Actigraphy

Wrist-worn actigraphs (AX3 device, Axivity, purchased ‘off-the-shelf’ and used within its indication), will be distributed to participants who will be requested to wear them for 7 days after the baseline visit. The 7-day assessment period can commence up to 3 days after the baseline visit to allow for any delay in shipment.

### Remote cognitive assessment

A neuropsychological battery using the Cognitron cognitive testing platform^26^ will be employed to gather data remotely. The battery will consist of executive function (Verbal Reasoning), attention (Simple Reaction Time, Choice Reaction Time, Digit Vigilance), working Memory (Paired Associate Learning, Self-Ordered Search, Digit Span) and episodic memory (Delayed Word Recognition) tasks. Eligible participants will be required to complete sessions of testing using a PC or a tablet on three consecutive days in the week after the baseline visit.

Once scanning and remote assessment procedures are complete, randomised participants initiate treatment with 3 mg oral semaglutide/placebo once daily and follow a 8-week dose escalation regimen until reaching the treatment dose of 14 mg oral semaglutide/placebo once daily. Participants to take the drug with half a glass of water and (i) to not split, crush or chew the tablet, (ii) to take it in the morning before any oral intake, (iii) not to eat, drink or take any other medication for 30 min after administration. Participants should remain on the 14 mg dose level until the end of treatment visit, but treatment interruptions are allowed, for example, if there are issues with poor tolerability or treatment emergent adverse events (AEs). Unscheduled visits are arranged as required in instances where a change in dose is necessitated to dispense a lower dose of the investigational medicinal product (IMP). If participants are unable to tolerate the IMP despite dose reductions or interruptions, the IMP is discontinued permanently. In this instance, participants should continue to follow the trial schedule without being withdrawn from the trial. Unblinding is possible in a medical emergency through the software employed for randomisation; unblinding will result in withdrawal from the study.

### Follow-up visits

Participants will attend visits at weeks 4, 8, 26 and 39 to monitor safety (emergence of AEs, changes to questionnaires relevant to health anxiety, depression and distress) and obtain blood for research purposes.

### Final visit (week 52)

The study will close out with a final visit at which all assessments from the baseline visit will be repeated: in-clinic cognitive testing (ACE-III, CANTAB) and EQ-5D-5L will be performed and scanning (PET Tau, PET TSPO and MRI) will be arranged. Participants will complete a final period of remote cognitive testing and actigraphy monitoring in the week before the final visit.

### Follow-up (week 57)

The study team will follow participants up with a telephone call to determine if any AEs have occurred in the period since discontinuing the medication.

### Total radiation exposure

The maximum total radiation protocol dose (TRPD) from this study is 28.4 mSv. For comparison, the average annual natural background radiation dose in the UK is 2.7 mSv. The TRPD incurred in this study can be compared with approximately 12 years of natural background radiation exposure. The risk from exposure to ionising radiation is the induction of fatal cancers and, assuming a risk for the UK population of both sexes for ages 18–64 years of 5% per Sievert (Documents of the National Radiological Protection Board Vol 4, No 4, 1993), the additional lifetime risk of inducing a fatal cancer in a healthy individual is approximately 1 in 704 from a dose of 28.4 mSv. This should be compared with the natural incidence rate for cancer in the UK, which is one in three.

### Sample size and power considerations

The proportion of participants with T2D will be limited to 30% to minimise the possible impact of any diabetes-specific effects of semaglutide. Table 2 shows 12 power estimates for 3 possible tau PET change effect sizes (based on the difference in mean 1-year change in tau accumulation between the semaglutide and placebo treatment groups) and two alpha (type 1 error) values, assuming 3.6 individuals need to be scanned to identify 1 amyloid-positive individual, 10% of the total participants recruited will be excluded for reasons unrelated to use of TSPO ligand, 10% have a genetic variant that precludes use of TSPO ligand and 14% potentially lost to follow-up. The study will therefore inform the size of the effect of semaglutide on tau PET accumulation rates for future confirmatory trials.

**Table 2:** Sample size estimations

Total number scanned for amyloid=316Number randomised=88Number of completers=75 (=88×0.86)	Power estimate* assuming tau PET mean annual change (SD) of 0.05 (0.04)† when untreated		Power estimate* assuming tau PET mean annual change (SD) of 2.01 (2.97)‡ when untreated	
One-year change from baseline in mean tau accumulation with semaglutide	Alpha 0.05	Alpha 0.10	Alpha 0.05	Alpha 0.10
20% lower compared with placebo. Effect differences of 0.01 and 0.402, respectively	19.7%	29.9%	9.2%	16.0%
30% lower compared with placebo. Effect differences of 0.015 and 0.603, respectively	38.1%	50.8%	14.6%	23.4%
40% lower compared with placebo. Effect differences of 0.02 and 0.804, respectively	59.8%	71.6%	22.3%	33.0%

316 individuals need to be screened to randomise 88 participants, with 75 participants completing the study, assuming a 14% dropout rate.

* Power calculation is based on a two-sample t-test assuming equal variance.

† Hanseeuw, BJ *et al*.^32^ 2019 PMID: 31157827.

‡ Whittington A, Gunn R.^33^ 2021 PMID: 33517326.

### Statistical analysis

A description of the planned statistical analysis can be found in the Statistical Analysis Plan (online supplemental appendix 1).

## Ethics and dissemination

ISAP was approved by the West Midlands—Edgbaston Research Ethics Committee (22/WM/0013). Any amendments are communicated by the trial team to the relevant parties. Dissemination of the study results will take place through peer-reviewed publications and scientific meeting presentations. Access to data for exploratory analyses and approval of publications, including authorship, will be subject to approval by the Trial Steering Committee.

## Key issues in trial design

### Participant stratification

The goal of the ISAP trial is to examine the impact of GLP-1 receptor agonism in predementia AD compared with placebo. Evidencing abnormal cortical amyloid using either PET or CSF is currently the most widely accepted method for establishing that an individual is on the AD pathophysiology continuum irrespective of the presence of clinical signs and symptoms.^27 28^ On this basis, amyloid positivity is a standard criterion for inclusion in AD-modifying treatments trials.^29^ Amyloid positivity has been demonstrated to predict conversion to AD dementia, both in those with a degree of cognitive impairment^30^ as well as asymptomatic individuals.^31^ Amyloid positivity also increases the rate at which pathology more tightly correlated with neurodegeneration, such as tau, accumulates.^32 33^ The established position of amyloid positivity as AD continuum biomarker and its impact on tau accumulation guided us in our choice of inclusion criterion. The decision to focus on PET-based testing was driven by a patient and public consultation in the study design stages which showed a preference for PET over lumbar punctures for potential participant

A major issue faced by trials relying on amyloid positivity is how to minimise the number of people excluded because of negative amyloid status when tested. A strategy of relying solely on age is high risk as a minority of cognitively unimpaired individuals are amyloid positive.^34^ *APOE* ε4 allele carriership is the most reliable predictor of abnormal amyloid load, increasing the likelihood by twofold to threefold regardless of the age group.^34^ Having MCI increases the risk substantially, whereby 27%–71% are amyloid positive and again this risk is mediated by *APOE* ε4 carriership.^34^ Efforts to further improve the prediction in cognitively unimpaired individuals have led to predictive models that include a variety of dementia risk-related data.^35 36^ For the purposes of the ISAP trial, we opted for a pragmatic risk-stratification method based on the type of data available through electronic research registers in the UK (PROTECT cohort, https://www.protectstudy.org.uk/^37^ and Dementias Platform UK Great Minds^38^): diagnosis of MCI, APOE4 carriership, family history of dementia in a first-degree relative, age and sex. We estimated that through this stratification method, we would need to scan 3.6 individuals to identify 1 amyloid-positive case.

### Efficacy biomarkers

Evidencing the effects of novel therapeutics in clinically silent or minimally symptomatic individuals is a major challenge for AD research. Various biomarkers are under investigation as surrogates of treatment response in prodromal AD.^39^ Of these, tau PET has the best evidence—it co-localises with neurodegeneration,^40^ predicts cognitive status^41^ and mirrors the clinical phenotype.^42^ In contrast, the site and extent of amyloid deposition does not correlate with neurodegeneration.^41 43^ In a head-to-head comparison versus MRI and amyloid PET, tau PET was shown to be the strongest predictor of cognitive decline and neurodegeneration in both cognitively impaired and healthy individuals.^44^ For this reason, it is currently the biomarker of choice for tracking treatment effects across the AD spectrum.^45^

While well validated, tau PET is limited in its utility for large-scale clinical trials because of its cost, invasiveness and reliance on infrastructure.^45^ Future research efforts therefore are directed towards identifying more scalable biomarkers. Plasma biomarkers of AD are in prime position to act as surrogates for developing AD pathology through their recently demonstrated strong associations.^46^ In fact, the plasma p-tau181 assay has been shown to become abnormal even before tau PET becomes abnormal which may make it even more suitable for early therapeutic signal detection,^47^ and similar results exist for Aβ42/Aβ40, p-tau217 and GFAP.^25 48^ Digitally derived measures may offer an alternative or complementary method for tracking effects in prodromal AD through the high density of data obtainable through passive and active cognitive function monitoring.^49^ To explore this important facet of prodromal AD trial methodology, in ISAP we implemented a high-density schedule of blood and digital biomarker sampling so that we can compare longitudinal variations in these biomarkers with the gold standard of tau PET.

### Amyloid disclosure to potential participants

Disclosure of increased risk for AD to cognitively normal individuals indicated by a positive amyloid result is inherent in preclinical AD trials. Whether this risk disclosure exposes individuals to psychological sequelae has been explored in previous research such as the anti-amyloid treatment in asymptomatic AD (A4) study.^50^ In that trial, cognitively normal individuals who tested amyloid positive were given a monoclonal antibody, solanezumab, to assess its impact on the rate of memory impairment progression. The A4 team found that individuals who learned that they have elevated amyloid did not experience an increase in depressive, anxiety or suicidality symptoms.^51^ Others have similarly reported low risk from psychological harm of amyloid disclosure.^52^ The process of conveying amyloid positivity however remains a key ethical consideration of preclinical AD trials and formal processes have been developed.^53^ We have followed these recommendations in the ISAP amyloid disclosure standard operating procedure; these will likely continue to evolve in future studies as the exact prognostic significance of amyloid PET and other AD biomarkers (eg, tau PET) in predementia adults becomes apparent.

## Conclusion

GLP-1 RAs are a promising drug class for both secondary prevention and treatment of dementia. Repurposing licensed compounds reduces the risk, cost and time to develop a new treatment and remains largely untested in AD. Studies that evaluate the potential mechanism of action of repurposed compounds in AD are a critical part of the development process as they can provide proof-of-concept, highlight novel treatment approaches and inform the appropriate power of future confirmatory trials. Through a randomised placebo-controlled trial design, ISAP aims to deliver on these opportunities for oral semaglutide while also providing a valuable biomarker dataset linking gold standard PET measures of cortical tau and neuroinflammation to promising plasma and digital biomarkers relevant to preclinical AD.

## supplementary material

10.1136/bmjopen-2023-081401 online supplemental file 1

10.1136/bmjopen-2023-081401 online supplemental file 2

## References

[1] Wilson DM, Cookson MR, Van Den Bosch L (2023). Hallmarks of neurodegenerative diseases. Cell.

[2] Jack CR, Bennett DA, Blennow K (2016). A/T/N: an unbiased descriptive classification scheme for alzheimer disease biomarkers. Neurology.

[3] Horby P, Lim WS, Group RC (2021). Dexamethasone in hospitalized patients with COVID-19. N Engl J Med.

[4] Ballard C, Aarsland D, Cummings J (2020). Drug repositioning and repurposing for alzheimer disease. Nat Rev Neurol.

[5] Nauck MA, Quast DR, Wefers J (2021). GLP-1 receptor agonists in the treatment of type 2 diabetes - state-of-the-art. Mol Metab.

[6] Medicines E, Compendium.

[7] Hunter K, Hölscher C (2012). Drugs developed to treat diabetes, liraglutide and lixisenatide, cross the blood brain barrier and enhance neurogenesis. BMC Neurosci.

[8] Nguyen T, Wen S, Gong M (2020). Dapagliflozin activates neurons in the central nervous system and regulates cardiovascular activity by inhibiting SGLT-2 in mice. Diabetes Metab Syndr Obes.

[9] Nørgaard CH, Friedrich S, Hansen CT (2022). Treatment with glucagon-like Peptide-1 receptor agonists and incidence of dementia: data from pooled double-blind randomized controlled trials and nationwide disease and prescription registers. Alzheimers Dement (N Y).

[10] Wium-Andersen IK, Osler M, Jørgensen MB (2019). Antidiabetic medication and risk of dementia in patients with type 2 diabetes: a nested case-control study. Eur J Endocrinol.

[11] Gejl M, Gjedde A, Egefjord L (2016). In Alzheimer’s disease, 6-month treatment with GLP-1 analog prevents decline of brain glucose metabolism: randomized, placebo-controlled, double-blind clinical trial. Front Aging Neurosci.

[12] Gejl M, Brock B, Egefjord L (2017). Blood-brain glucose transfer in alzheimer’s disease: effect of GLP-1 analog treatment. Sci Rep.

[13] Mullins RJ, Diehl TC, Chia CW (2017). Insulin resistance as a link between amyloid-beta and tau pathologies in alzheimer’s disease. Front Aging Neurosci.

[14] Marciniak E, Leboucher A, Caron E (2017). Tau deletion promotes brain insulin resistance. J Exp Med.

[15] Rodriguez-Rodriguez P, Sandebring-Matton A, Merino-Serrais P (2017). Tau hyperphosphorylation induces oligomeric insulin accumulation and insulin resistance in neurons. Brain.

[16] Sultan A, Nesslany F, Violet M (2011). Nuclear tau, a key player in neuronal DNA protection. J Biol Chem.

[17] Ittner LM, Ke YD, Delerue F (2010). Dendritic function of tau mediates amyloid-beta toxicity in alzheimer’s disease mouse models. Cell.

[18] Leng F, Edison P (2021). Neuroinflammation and microglial activation in alzheimer disease: where do we go from here. Nat Rev Neurol.

[19] Verma S, Bhatta M, Davies M (2023). Effects of once-weekly semaglutide 2.4 mg on c-reactive protein in adults with overweight or obesity (STEP 1, 2, and 3): exploratory analyses of three randomised, double-blind, placebo-controlled, phase 3 trials. EClinicalMedicine.

[20] Yoon G, Kim YK, Song J (2020). Glucagon-like Peptide-1 suppresses neuroinflammation and improves neural structure. Pharmacol Res.

[21] Livingston G, Huntley J, Sommerlad A (2020). Dementia prevention, intervention, and care: 2020 report of the lancet commission. Lancet.

[22] Holman RR, Bethel MA, Mentz RJ (2017). Effects of once-weekly exenatide on cardiovascular outcomes in type 2 diabetes. N Engl J Med.

[23] Rossing P, Bain SC, Bosch-Traberg H (2023). Effect of semaglutide on major adverse cardiovascular events by baseline kidney parameters in participants with type 2 diabetes and at high risk of cardiovascular disease: SUSTAIN 6 and PIONEER 6 post hoc pooled analysis. Cardiovasc Diabetol.

[24] Zetterberg H (2022). Biofluid-based biomarkers for alzheimer’s disease-related pathologies: an update and synthesis of the literature. Alzheimers Dement.

[25] Keshavan A, Wellington H, Chen Z (2020). Concordance of CSF measures of alzheimer’s pathology with amyloid PET status in a preclinical cohort: a comparison of lumipulse and established immunoassays. Alzheimers Dement (Amst).

[26] Hampshire A, Hellyer P https://www.cognitron.co.uk.

[27] Sperling RA, Aisen PS, Beckett LA (2011). Toward defining the preclinical stages of alzheimer’s disease: recommendations from the national institute on aging-alzheimer’s association workgroups on diagnostic guidelines for alzheimer’s disease. Alzheimers Dement.

[28] Jack CR, Bennett DA, Blennow K (2018). NIA-AA research framework: toward a biological definition of alzheimer’s disease. Alzheimers Dement.

[29] Rabinovici GD (2021). Controversy and progress in alzheimer’s disease - FDA approval of aducanumab. N Engl J Med.

[30] Sörensen A, Blazhenets G, Schiller F (2020). Amyloid biomarkers as predictors of conversion from mild cognitive impairment to alzheimer’s dementia: a comparison of methods. Alzheimers Res Ther.

[31] Donohue MC, Sperling RA, Petersen R (2017). Association between elevated brain amyloid and subsequent cognitive decline among cognitively normal persons. JAMA.

[32] Hanseeuw BJ, Betensky RA, Jacobs HIL (2019). Association of amyloid and tau with cognition in preclinical alzheimer disease: a longitudinal study. JAMA Neurol.

[33] Whittington A, Gunn RN (2021). Alzheimer’s disease neuroimaging I. Tau(IQ): a canonical image based algorithm to quantify tau PET scans. J Nucl Med.

[34] Jansen WJ, Ossenkoppele R, Knol DL (2015). Prevalence of cerebral amyloid pathology in persons without dementia: a meta-analysis. JAMA.

[35] Petersen KK, Lipton RB, Grober E (2022). Predicting amyloid positivity in cognitively unimpaired older adults: a machine learning approach using A4 data. Neurology.

[36] Calvin CM, de Boer C, Raymont V (2020). 'Prediction of alzheimer’s disease biomarker status defined by the 'ATN framework' among cognitively healthy individuals: results from the EPAD longitudinal cohort study'. Alzheimers Res Ther.

[37] PROTECT study.

[38] Koychev I, Young S, Holve H (2020). Dementias platform UK clinical studies and great minds register: protocol of a targeted brain health studies recontact database. BMJ Open.

[39] Koychev I, Lawson J, Chessell T (2019). Deep and frequent phenotyping study protocol: an observational study in prodromal alzheimer’s disease. BMJ Open.

[40] Jack CR, Dickson DW, Parisi JE (2002). Antemortem MRI findings correlate with hippocampal neuropathology in typical aging and dementia. Neurology.

[41] Giannakopoulos P, Herrmann FR, Bussière T (2003). Tangle and neuron numbers, but not amyloid load, predict cognitive status in alzheimer’s disease. Neurology.

[42] Ossenkoppele R, Schonhaut DR, Schöll M (2016). Tau PET patterns mirror clinical and neuroanatomical variability in alzheimer’s disease. Brain.

[43] Bennett DA, Schneider JA, Wilson RS (2004). Neurofibrillary tangles mediate the association of amyloid load with clinical alzheimer disease and level of cognitive function. Arch Neurol.

[44] Ossenkoppele R, Smith R, Mattsson-Carlgren N (2021). Accuracy of tau positron emission tomography as a prognostic marker in preclinical and prodromal alzheimer disease: a head-to-head comparison against amyloid positron emission tomography and magnetic resonance imaging. JAMA Neurol.

[45] Groot C, Villeneuve S, Smith R (2022). Tau PET imaging in neurodegenerative disorders. J Nucl Med.

[46] Janelidze S, Berron D, Smith R (2021). Associations of plasma phospho-tau217 levels with tau positron emission tomography in early alzheimer disease. JAMA Neurol.

[47] Moscoso A, Grothe MJ, Ashton NJ (2021). Time course of phosphorylated-tau181 in blood across the alzheimer’s disease spectrum. Brain.

[48] Ashton NJ, Janelidze S, Mattsson-Carlgren N (2022). Differential roles of Abeta42/40, P-Tau231 and P-Tau217 for Alzheimer’s trial selection and disease monitoring. Nat Med.

[49] Chinner A, Blane J, Lancaster C (2018). Digital technologies for the assessment of cognition: a clinical review. Evid Based Ment Health.

[50] Sperling RA, Rentz DM, Johnson KA (2014). The A4 study: stopping AD before symptoms begin. Sci Transl Med.

[51] Grill JD, Raman R, Ernstrom K (2020). Short-term psychological outcomes of disclosing amyloid imaging results to research participants who do not have cognitive impairment. JAMA Neurol.

[52] de Wilde A, van Buchem MM, Otten RHJ (2018). Disclosure of amyloid positron emission tomography results to individuals without dementia: a systematic review. Alzheimers Res Ther.

[53] Harkins K, Sankar P, Sperling R (2015). Development of a process to disclose amyloid imaging results to cognitively normal older adult research participants. Alzheimers Res Ther.

