# Supplementary material for: Protocol for a double-blind placebo-controlled randomised controlled trial assessing the impact of oral semaglutide in amyloid positivity (ISAP) in community dwelling UK adults
Source: BMJ Open. 2024 Jun 20;14(6):e081401. doi: 10.1136/bmjopen-2023-081401 (PMC11328662; doi:10.1136/bmjopen-2023-081401)
Supplement: online supplemental file 1 [file bmjopen-14-6-s001.pdf]

Local logo/letterhead

Participant identification number:

CONSENT FORM
Impact of Semaglutide in Amyloid Positivity (ISAP) study

Please initial each box if you agree

1	I confirm that I have read and understand the information sheet dated _______________ (version X.X) for the above study. I have had the opportunity to consider the information, ask questions and have had these answered satisfactorily.	
2	I understand that my participation is voluntary and that I am free to withdraw at any point, without giving any reason, without my medical care or legal rights being affected.	
3	I understand that I will only be included in the study if I am found to be suitable during the screening assessments.	
4	I have been advised about the potential risks associated with taking part in this research and have taken these into consideration before consenting to participate.	
5	I have been advised as to what I need to do for this research (especially with regard to semaglutide intake) and I agree to follow the instructions given to me.	
6	I understand that relevant sections of my medical notes and data collected during the study may be looked at by members of the site study team, monitors and designated individuals from the University of Oxford, the funder (Novo Nordisk), regulatory authorities and the participating Universities/NHS Trust(s), where it is relevant to my taking part in this study. I give permission for these individuals to have access to my records.	
7	I agree to my General Practitioner being informed of my participation in the study and of any abnormal results arising during the study that may be of clinical relevance. I agree to my GP providing researchers with health information relevant to my participation in the study in the event that I stop attending follow-up visits.	
8	I understand that the MRI/PET scans for the study are research scans that are not useful for medical diagnosis, and that scans are not routinely looked at by a doctor. If a concern is raised about a possible abnormality on my scan, I will only be informed if a doctor thinks it is medically important such that the finding has clear implications for my current or future health.	

Local logo/letterhead

9 I agree for my personal and health data (such as my name, date of birth, NHS number, address) and participant code to be shared with members of the Imperial College London team performing genetic testing on my blood sample as well as imaging. I understand that the purpose of this is so that they can safely identify me for the purposes of the scans. I understand that the form with my personal information will be retained at Imperial College London for the duration of the study but the sample used for genetic testing will be destroyed after analysing. I understand that the results will be recorded on an electronic patient record and sent to the study site team.

10 I understand that the coded information collected about me will be used to support other research in the future, and may be transferred to, and stored at, a destination outside the UK and the European Economic Area for analysis. I understand this research may involve commercial organisations.

11 I agree for my voice responses from the tablet memory tasks to be recorded and analysed for the purposes of the study.

12 I agree to donate blood samples and consider these samples a gift to the University of Oxford and understand I will not gain any direct personal or financial benefit from them.

13 I understand and agree that my samples will be used in research aimed at understanding the genetic influences on disease and that the results of these investigations are unlikely to have any implications for me personally.

14 I agree for my blood samples to be used in future research, here or abroad, which has ethics approval. I understand this research may involve commercial organisations.

15 I agree to take part in the above study.

Optional:	I agree for my contact details to be kept in a secure database for the purpose of contacting me about future ethically approved studies. I understand that agreeing to be contacted does not oblige me to participate in any further studies.	Yes<br>No	
Optional	If recruited through an existing research register, I agree for my results from the baseline ISAP assessments to be shared with the referring researchers.	Yes<br>No<br>N/A	

dd / mmm / yyyy

Name of Participant | Date | Signature

dd / mmm / yyyy

Name of person taking consent | Date | Signature

When completed: 1 for participant (copy); 1 for researcher site file (original)
