# Supplementary material for: Protocol for a double-blind placebo-controlled randomised controlled trial assessing the impact of oral semaglutide in amyloid positivity (ISAP) in community dwelling UK adults
Source: BMJ Open. 2024 Jun 20;14(6):e081401. doi: 10.1136/bmjopen-2023-081401 (PMC11328662; doi:10.1136/bmjopen-2023-081401)
Supplement: online supplemental file 2 [file bmjopen-14-6-s002.pdf]

E-Sign ID: 54e08cc4-0d2e-4185-9d84-2287f4ade13f

Impact of Semaglutide in Amyloid Positivity (ISAP) Study

Statistical Analysis Plan

Version 1.0

Date: 20th October 2023

Aligned with protocol version: 7.0, 28th August 2023

Ethics Ref: 22/WM/0013

IRAS Project ID: 300550

EudraCT Number: 2021-003328-34

Novo Nordisk Trial ID: NN6535-4887

Oxford University PID: 15869

Contents

LIST OF INVESTIGATORS, SPONSOR AND FUNDER 6
LIST OF AUTHORS AND REVIEWERS: 7
1.0 Purpose and Scope of the Statistical Analysis Plan 8
2.0 Introduction 8
2.1 Background and Rationale 8
2.2 Objectives of the Trial 9
2.2.1 *Primary objective* 9
2.2.2 *Key Exploratory objectives* 9
2.3 Trial Design 9
2.4 Eligibility 9
2.4.1 *Trial participants* 9
2.4.2 *Inclusion criteria* 10
2.4.3 *Exclusion criteria* 10
2.5 Randomisation 11
2.6 Sample Size 11
2.7 Treatments 13
2.8 Blinding of Investigational Medicinal Product (IMP) 13
2.9 Endpoints 13
2.9.1 *Primary outcome measure* 13
2.9.2 *Exploratory outcome measures* 14
3.0 Analysis Populations 14
3.1 Target Population 14
3.2 Trial Population 14
3.3 Trial Sample 15
4.0 General Considerations 15
4.1 Timing of Analyses 15
4.2 Stopping Rules 15
4.3 Baseline Stratifiers, Baseline Outcomes and Subgroup Analyses 15
4.4 Level of Significance 16
5.0 Descriptive Analysis 16
5.1 Recruitment and Follow-up Patterns 16
5.2 Baseline Characteristics 17
5.3 Outcomes 17
5.4 Aherence to Treatment and Lost to Follow-up and Pattern of Missingness 18
6.0 Comparative Analyses 18

6.1 Analysis of Primary Outcome 18
6.2 Analysis of Secondary and Exploratory Outcomes 18
6.3 Analysis of Safety Data 19
7.0 Software 19
7.1 Data Management 19
7.2 Statistical Analysis 19
8.0 References 19
9.0 Approval 20
10.0 Appendix 22

ABBREVIATIONS:

Aβ40	Amyloid beta 40
Aβ42	Amyloid beta 42
ACE-3	Addenbrooke's Cognitive Assessment
AD	Alzheimer's disease
AE	Adverse event
ANCOVA	Analysis of Covariance
AR	Adverse Reaction
CANTAB	Cambridge Neuropsychological Test Automated Battery
CDR	Clinical Dementia Rating
CI	Confidence Interval
CKD-EPI	Chronic Kidney Disease Epidemiology Collaboration
CNS	Central Nervous System
CSF	Cerebrospinal fluid
DAS	Data Specification
DMC	Data Monitoring Committee
DTU	Diabetes Trial Unit
ECG	Electrocardiogram
EDC	Electronic Data Capture
eGFR	Estimated Glomerular Filtration Rate
EQ-5D-5L	EuroQol-5 Dimension-5 Level
EudraCT	European Union Drug Regulating Authorities Clinical Trial Database
GCP	Good Clinical Practice
GFAP	Glial fibrillary acidic protein
GLP-1 RA	Glucagon-like peptide-1 receptor agonist
GP	General Practitioner
HbA1c	Glycated haemoglobin
HR	Hazards ratio
ICH	International Conference on Harmonisation
IMP	Investigational Medicinal Product
IQR	Interquartile Range
ISAP	Impact of Semaglutide in Amyloid Positivity
ITT	Intention To Treat

MAR	Missing at Random assumption
MCI	Mild Cognitive Impairment
mITT	Modified Intention To Treat
MM	Mixed Model
MODY	Maturity-onset diabetes of the young
MRC BSU	MRC Biostatistics Unit
MRI	Magnetic resonance imaging
NFL	Neurofilament light
NHS	National Health Service
PET	Positron emission tomography
PPS	Per-Protocol Set
p-tau181	Phosphorylated tau181
REC	Research Ethics Committee
RS	Randomised Set
SAE	Serious Adverse Event
SAP	Statistical Analysis Plan
SAR	Serious Adverse Reaction
SD	Standard Deviation
SOP	Standard Operating Procedure
SUSAR	Suspected Unexpected Serious Adverse Reactions
SUVR	Standardised Uptake Value Ratio
T2DM	Type 2 diabetes mellitus
TS	Treated Set
TSC	Trial Steering Committee
TSPO	Translocator protein

LIST OF INVESTIGATORS, SPONSOR AND FUNDER

Investigators

Dr Ivan Koychev, Chief Investigator, Department of Psychiatry, University of Oxford. ivan.koychev@psych.ox.ac.uk

Professor Amanda Adler, Diabetes Trials Unit, University of Oxford. amanda.adler@dtu.ox.ac.uk

Professor Rury Holman, Diabetes Trials Unit, University of Oxford. rury.holman@dtu.ox.ac.uk

Dr Paul Edison, Department of Brian Sciences, Imperial College London. paul.edison@imperial.ac.uk

Dr Brian Tom, MRC Biostatistics Unit, University of Cambridge. brian.tom@mrc-bsu.cam.ac.uk

Dr Joe Butchart, Medical School, Royal Devon and Exeter NHS Foundation Trust. joseph.butchart@nhs.net

Professor Adam Hampshire, Department of Brain Sciences, Imperial College London. a.hampshire@imperial.ac.uk

Dr Charles Marshall, Tower Hamlets, East London NHS Foundation Trust charles.marshall@qmul.ac.uk

Dr Elizabeth Coulthard, Clinical Neuroscience, North Bristol NHS Trust. Elizabeth.Coulthard@bristol.ac.uk

Professor Henrik Zetterberg, UCL Queen Square Institute of Neurology, University College London. h.zetterberg@ucl.ac.uk

Dr Francesca Cormack, Cambridge Cognition Ltd, Cambridge. Francesca.cormack@camcog.com

Dr Catherine Mummery, National Hospital for Neurology and Neurosurgery, University College London Hospitals. cath.mummery@nhs.net

Dr Nazar Mansour, Central and North West London NHS Foundation Trust. n.mansour@nhs.net

Dr Robert Lawrence, South West London & St George's Mental Health NHS Trust. Robert.Lawrence@swlstg.nhs.uk

Dr Sabari Muthukrishnan, Kingshill Research Centre, Avon and Wiltshire Mental Health Partnership NHS Trust. s.muthukrishnan1@nhs.net

Dr Simon Vann-Jones, Cornwall Partnership NHS Foundation. s.vannjones@nhs.net

Dr Ben Underwood, Department of Psychiatry, University of Cambridge.
Ben.Underwood@cpft.nhs.uk ben.underwood@cpft.nhs.uk

Sponsor

University of Oxford, Research Governance, Ethics and Assurance Team, Joint Research Office, 1st floor, Boundary Brook House, Churchill Drive, Headington, Oxford OX3 7GB

Funder

Novo Nordisk A/S, Novo Allé 1, 2880 Bagsvaerd, Denmark.

LIST OF AUTHORS AND REVIEWERS:

Authors

Dr Brian D M Tom, MRC Investigator, Programme Leader, MRC Biostatistics Unit, University of Cambridge. brian.tom@mrc-bsu.cam.ac.uk

Reviewers

Ruth Coleman, Senior Research Statistician, Diabetes Trials Unit, University of Oxford. ruth.coleman@dtu.ox.ac.uk

Rury Holman, Emeritus Director, Diabetes Trials Unit, University of Oxford. rury.holman@dtu.ox.ac.uk

Amanda Adler, Director, Diabetes Trials Unit, University of Oxford. Amanda.adler@dtu.ox.ac.uk

1.0 Purpose and Scope of the Statistical Analysis Plan

The purpose of this document is to set out the study objectives and hypotheses, the proposed presentation, analytical approaches and procedures necessary for reporting results for the main trial paper(s) of the multi-centre randomised, double-blind, controlled trial on the Impact of Semaglutide in Amyloid Positivity (ISAP).

As there can typically be multiple analytic approaches and strategies for addressing a hypothesis, there is the potential for different results to be realised from the use of alternative approaches, methods, outcome definitions and data that may be involved. Therefore the results reported in the main trial paper(s) will follow the strategy set out in this Statistical Analysis Plan (SAP); developed prior to the availability of follow-up data and finalised before database lock. Changes within any subsequent version of the SAP prior to analysis will be dated, with the basis/justification for these changes recorded.

The rationale, decision and strategy to be followed, as described in this SAP, will comply with the study protocol, Good Clinical Practice (GCP) guidelines, the statistical guidance/principles set out in the International Conference on Harmonisation of Technical Requirements for Registration of Pharmaceuticals for Human Use (ICH) guidelines E9 – Statistical Principles for Clinical Trials[1], the CONSORT statement[2] for reporting trials and other statutory and regulatory requirements as appropriate.

Note that there may be possible additional analytic decisions that need to be taken after database lock (e.g. based on viewing the observed distribution of the follow-up data, prior to the trial arm being made available). Any deviations from the SAP will be described and justified and will be appended to the SAP for record purposes.

Note finally that any post hoc analyses of a more exploratory nature or not directly related to the main aims of the trial are not bound by this plan.

2.0 Introduction

2.1 Background and Rationale

Alzheimer's Disease (AD), characterised by synaptic dysfunction and neurodegeneration, is thought to be triggered by the sequential accumulation of amyloid plaques and neurofibrillary tangles which are aggregates of hyperphosphorylated tau proteins[3]. This process is understood to begin decades before first symptoms, with supra-threshold levels of cortical amyloid accumulation deemed triggering the condition; such 'amyloid positivity' as evidenced by PET scans or cerebrospinal fluid (CSF) assays in cognitively healthy individuals is now considered diagnostic of their being in the preclinical stages of AD[4]. Treatments designed to directly reduce accumulations of these abnormal proteins have so far not yielded beneficial results. Currently approved AD therapies are hence limited to symptomatic treatment which are of limited benefit. Developing an effective disease modifying therapy for AD therefore remains as one of the key unmet needs of modern medicine owing to the prevalence of this

condition and the associated disability, societal costs and increased mortality. Dementia and AD have been the number one cause of death in the UK in 2021.[5]

Glucagon-like peptide-1 receptor agonists (GLP-1 RAs) may offer novel mechanisms to delay or even prevent neurotoxicity in individuals at-risk for AD. Studies in preclinical models have shown that administrating a GLP-1 RA is associated with a reduction in the effect of neurotoxic agents, decreases in the extent of AD protein and neuroinflammatory burden, and improved memory function. Pooled data from three double-blind randomised placebo-controlled studies in patients with type 2 diabetes mellitus (T2DM) demonstrated, in exploratory analyses, a reduction in the risk of developing dementia in patients treated with a GLP-1 RA, compared with placebo, over a 3.6-year follow-up period (Hazards Ratio (HR) 0.47, 95%CI 0.25-0.86). Additionally, analysis of the REWIND trial showed that dulaglutide, another GLP-1RA, reduced cognitive impairment in T2DM participants. Internal analysis by Novo Nordisk on the TRUVEN Medicare Supplemental and Coordination of Benefits Database found that more than 2 years of GLP-1 RA exposure resulted in an approximately 30% decrease in the risk of dementia, compared with no GLP-1 RA exposure (HR 0.69, 95%CI 0.57-0.85). However, the mechanisms for the potential disease modifying action of GLP-1 RAs with regard to dementia remains unclear.

In ISAP, we aim to explore possible mechanisms underlying the potential disease modifying effects of semaglutide, in a group of individuals with preclinical AD defined as being amyloid positive on PET and having no diagnosis of dementia.

2.2 Objectives of the Trial

2.2.1 *Primary objective*

The primary objective of the trial is to explore the disease modifying effects of oral semaglutide on tau accumulation rates (determined by PET) in preclinical AD over 52-week follow-up.

2.2.2 *Key Exploratory objectives*

To assess the effects of oral semaglutide on neuroinflammation (as determined by PET and blood assays), plasma blood biomarkers of AD pathology, cognitive changes, safety, neurodegeneration biomarkers (as determined by MRI and blood assays), health-related quality of life, diurnal activity variation (as determined by wrist-worn actigraphy) in preclinical AD over 52-week follow-up.

2.3 Trial Design

This is a multi-centre, randomised, double-blind, placebo-controlled, parallel-group, superiority trial of oral semaglutide in preclinical AD participants with follow-up over 52 weeks.

2.4 Eligibility

2.4.1 *Trial participants*

Amyloid-positive healthy volunteers of both sexes aged 55 years and over with no or minimal Mild Cognitive Impairment (MCI), as determined by a Clinical Dementia Rating (CDR) scale score ≤0.5, who speak English fluently.

2.4.2 *Inclusion criteria*

- Participant is willing and able to give informed consent for participation in the trial.
- Male or female, aged 55 years or above.
- Amyloid-positivity as evidenced by PET.
- Fluent English speaker as assessed by the Investigator.
- In the Investigator's opinion, is able and willing to comply with all trial requirements.
- Willing to allow their General Practitioner (GP), if appropriate, to be notified of participation in the trial.
- Clinical Dementia Rating (CDR) ≤0.5.
- An informant that is available to the research team for the purposes of the CDR scoring.

2.4.3 *Exclusion criteria*

- Diagnosis of dementia.
- Treatment with a GLP-1 RA: current or in the past 6 months
- Women who are pregnant, breastfeeding or of childbearing potential (see Appendix D of Protocol for definition).
- People with type 1 diabetes mellitus, secondary diabetes, or maturity-onset diabetes of the young (MODY).
- People with T2DM who have pre-proliferative or proliferative diabetic retinopathy, or diabetic maculopathy.
- People with T2DM if the cap of 30% of participants with T2DM randomised has been met.
- Poorly controlled T2DM defined as HbA1c ≥10% (≥86 mmol/mol).
- Evidence of severe renal impairment or an estimated glomerular filtration rate (eGFR) derived from serum creatinine (using the simple CKD-EPI formula) of <30 mL/min/1.73 m^2.
- Evidence of hepatic cirrhosis as assessed by medical history.
- A psychiatric condition which in the opinion of the investigator may affect the safety of the participant or the outcomes of the study.
- Any contraindication for MRI or PET scans, including but not limited to: MR-incompatible pacemakers, pregnancy, aneurysm clip, implanted neural stimulator, implanted cardiac pacemaker or auto-defibrillator, cochlear implant, ocular foreign body, recent carotid stent, CSF shunt, other implanted medical device, e.g., Swan Ganz catheter, insulin pump, as assessed by a standard pre-MRI questionnaire.
- Participant with a life expectancy of less than 6 months.
- Currently enrolled in another investigational device or drug study, or less than 30 days between randomisation and ending another investigational device or drug study or receiving other investigational treatment(s). Patients participating in a purely observational trial will not be excluded.
- Presence or history of malignant neoplasm (other than basal or squamous cell skin cancer, in-situ carcinomas of the cervix, or in situ prostate cancer) within 5 years prior to the day of screening.

- Lack of access to a suitable digital technology to allow remote cognitive testing (PC or tablet connected to the internet).
- Significant eye or hearing impairment that in the opinion of the investigator may affect study procedures.
- People with the low-affinity binding variant of the rs6971 allele of the TSPO gene.
- Known or suspected hypersensitivity to trial product or related products.
- Poor venous access or other contraindications that would make blood sampling difficult.
- Participant that in the view of the investigator will experience significant distress in the event of a positive amyloid status disclosure. Such individuals will not undergo amyloid screening.
- Diabetic individuals treated with sulphonylureas or insulin where dose adjustment as described in protocol is not possible for whatever reason.
- Individuals with significant radiation exposure in the past year for whom in the opinion of the investigator the additional trial-related exposure will result in an unacceptable risk.

2.5 Randomisation

At their Randomisation Visit (Visit 2), study participants who fulfil all the inclusion criteria (see 2.4.2) and violate none of the exclusion criteria (see 2.4.3) will be assigned a unique randomisation number that will allow subsequent identification of their randomised treatment group allocation.

Randomisation numbers will be allocated to eligible participants by the ISAP Electronic Data Capture (EDC) platform to assign semaglutide or matching placebo in an overall 1:1 allocation ratio. This assignment will be performed using a computerised procedure with minimisation (adaptive stratified sampling) to maintain balance between treatment groups based on 3 factors: T2DM (Yes/No), MCI (defined as CDR score 0.5) (Yes/No) and Site to maintain balance between treatment groups. No participant replacement will be allowed.

2.6 Sample Size

As a formal sample size estimation is infeasible in the absence of informative prior studies, the number of participants to be studied reflects the available funding. Table 1 shows twelve estimates of power for three possible tau PET change effect sizes and two alpha (Type 1 error) values. These calculations are based on the assumptions that there will be 10% of the total participants recruited (i.e. 39 of the estimated 390 participants recruited) excluded for reasons not related to use of TSPO ligand, followed by a further 10% of the remaining (i.e. 35 of 351) excluded due to having a genetic variant that precludes use of TSPO ligand. Additionally, we assume that 3.6 individuals will be needed to be scanned to identify one amyloid positive individual and that there will be 14% potential lost to follow-up.

**Total number scanned for amyloid = 316<br>Number randomised = 88<br>Number of completers = 75<br>(=88*0.86)**	**Power levels assuming tau PET mean annual change (SD) of 0.05 (0.04)***	**Power levels assuming tau PET mean annual change (SD) of 2.01 (2.97)†**

One-year change from baseline in mean tau accumulation with semaglutide	Alpha 0.05	Alpha 0.10	Alpha 0.05	Alpha 0.10
20% lower compared with placebo	19.7%	29.9%	9.2%	16.0%
30% lower compared with placebo	38.1%	50.8%	14.6%	23.4%
40% lower compared with placebo	59.8%	71.6%	22.3%	33.0%

* *[6]Hanseeuw, BJ et al. 2019 PMID: 31157827;* † *[7]Whittington A, Gunn R. 2021 PMID: 33517326*

Table 1: Power calculations for a number of scenarios

2.7 Treatments

The trial is randomised with 2 arms with equal allocation of eligible participants in a 1:1 ratio to treatment or placebo.

Randomised participants will initiate treatment with 3 mg oral semaglutide/placebo once daily and follow a 4-week dose escalation regimen until reaching the treatment dose of 14 mg oral semaglutide/placebo once daily. Participants should remain on the 14 mg dose level until the end of treatment visits, but down titration and treatment restarts will be permitted where appropriate.

2.8 Blinding of Investigational Medicinal Product (IMP)

The placebo tablets will be identical in visual appearance to the IMP semaglutide tablet. Furthermore, the appearance of the semaglutide tablets will be the same irrespective of their dose.

2.9 Endpoints

2.9.1 *Primary outcome measure*

The primary endpoint is the annualized change in cortical PET tau standardised uptake value ratio (SUVR), derived from the difference in PET tau SUVR taken at the baseline and 52-week visits compared between treatment groups.

2.9.2 *Exploratory outcome measures*

- Annualised change in translocator protein (TSPO) PET SUVR over 52 weeks from baseline.
- Repeatedly measured plasma glial fibrillary acidic protein (GFAP) protein levels at screening, baseline, weeks 4, 8, 26, 39 and 52.
- Repeatedly measured plasma AD biomarkers (p-tau181, Aβ42/40 ratio) at screening, baseline, weeks 4, 8, 26, 39 and 52.
- Repeatedly measured pen and paper cognitive test (Addenbrooke's Cognitive Assessment (ACE-III)) scores at baseline, weeks 26 and 52.
- Repeatedly measured computerised in-clinic cognitive battery (CANTAB) at baseline, weeks 26 and 52.
- Repeatedly measured remote cognitive battery (Cognitron)[1] at baseline, weeks 26 and 52.
- Presence of Adverse Events (AEs), Serious Adverse Events (SAEs), Adverse Reactions (ARs), Serious Adverse Reactions (SARs) and Suspected Unexpected Serious Adverse Reactions (SUSARs) collected at baseline, weeks 4, 8, 26, 39, 52 and follow-up call.
- Annualized change in MRI-based neurodegeneration biomarkers (hippocampal volume) over 52 weeks from baseline.
- Repeatedly measured plasma neurofilament light (NFL) at screening, baseline, weeks 4, 8, 26, 39 and 52.
- Change in depression (CES-D) and anxiety (HAI) scores over 52 weeks from baseline
- Levels of distress at AD risk disclosure at weeks 26 and 52 (Impact of Genetic Testing for Alzheimer's disease scale)
- Change in quality of life as measured by EQ-5D-5L over 52 weeks from baseline.
- Change in level and pattern of activity and circadian rhythms as measured using wrist-worn actigraphy[1] (functional data) at baseline and 52 weeks.

Exact details on the list of data to be collected and the scheduling of procedures to be performed over the study period can be found in Appendix B of the ISAP Trial Protocol.

3.0 Analysis Populations

3.1 Target Population

The *target population*, to which inferences from the end of this trial are intended to generalise, is the population with pre-clinical AD participants aged 55 years and over.

3.2 Trial Population

The trial population, from which the study sample is drawn, is further defined to be participants aged 55 years or older in whom a positive screening based on clinical information for

[1] Actigraphy and remote cognitive testing will be completed in the week before randomization (baseline) and week before the final visit (52-week).

potentially higher risk of amyloid positivity and subsequent positive baseline amyloid beta PET imaging scan and meeting other defined trial eligibility criteria.

3.3 Trial Sample

The achieved trial sample comprises those participants who consent to participate and are actually randomised into this trial. These participants, whether treated or not, comprise the Randomised Set (RS). The Treated Set (TS) will include all subjects from the RS who were documented to have received at least one dose of study drug or placebo. This TS is the (modified) Intention To Treat (mITT) population.

The per-protocol set (PPS) includes all patients from the TS who provide evaluable data at baseline and on-treatment for the primary endpoint and are not affected by protocol violations (including non-compliance – evidence for less than 80% of all doses being taken) relevant to the statistical evaluation of the primary endpoint.

4.0 General Considerations

4.1 Timing of Analyses

The statistical analyses will be performed by the MRC Biostatistics Unit (MRC BSU), University of Cambridge and validated by an independent statistician. The main analyses will be on the securely, transferred, finalised and blinded data from the locked database, having been documented as meeting the cleaning and approval requirements of the Diabetes Trial Unit (DTU), University of Oxford Standard Operating Procedures (SOPs) and after the finalisation and approval of this SAP document.

No interim analyses are planned.

4.2 Stopping Rules

The trial may be prematurely discontinued by the Sponsor, Chief Investigator (CI) or Regulatory Authority on the basis of new safety information or for other reasons given by the Data Monitoring or Trial Steering Committee (DMC or TSC), the Regulatory Authority or Ethics Committee concerned. If the trial is prematurely discontinued, active study participants will be notified, and no further participant data collected. The Competent Authority and Research Ethics Committee (REC) will be informed within 15 days of the early termination of the trial.

The DMC will review the accumulating safety data every 6 months, or as deemed necessary. Interim safety analyses conducted by the DMC will be performed by the DTU Independent Statistician under the aegis of the DMC statistician and are detailed in a separate DMC SAP. There are no formal stopping rules. DMC guidance will be based on a per-protocol approach analysis, with an intention-to-treat approach used for sensitivity analyses. A statistical guideline of significance level for all-cause mortality at $p<0.01$ will be considered as evidence of recommending early stopping for safety.

4.3 Baseline Stratifiers, Baseline Outcomes and Subgroup Analyses

In the primary analyses, to assess whether there is a meaningful difference between the two arms in the annualized change in cortical PET tau SUVR, adjustments will be made for baseline variables used to minimise over in treatment allocation (i.e. T2DM status, MCI status and Site).

The corresponding baseline measure for a continuous outcome is often predictive of the outcome (and change in outcome) at follow-up, whereas standard errors of statistics derived from binary outcomes vary little with the prevalence to offer gains in precision from adjustment of baseline but expend degrees of freedom. Therefore, the corresponding baseline outcome will be an additional covariate when modelling continuous outcomes.

No subgroup analyses are planned.

4.4 Level of Significance

The primary hypothesis will be assessed using a two-tailed test at the 5%-level of significance. Specific *a priori* non-hierarchical secondary hypotheses will be evaluated using a two-sided 2.5%-level of significance. Other tests will be considered exploratory. However, 95% confidence intervals will be constructed around treatment effects. No adjustments will be made for multiple testing.

5.0 Descriptive Analysis

5.1 Recruitment and Follow-up Patterns

The flow of participants through the trial from enrolment to analysis will be in accordance with CONSORT guidelines[2] and will be similar to the flow diagram in Figure 1 which provides information about how the trial was conducted; reporting enrolment, allocation, follow-up and analysis of patients involved in the trial. This will include for each group, the number of participants randomised, the intention to treat (mITT) population, the numbers followed-up to be in the analysis of the primary outcome as well as the numbers and reasons missing data (e.g. withdrawn from treatment, lost to follow-up, died during the study) after randomisation.

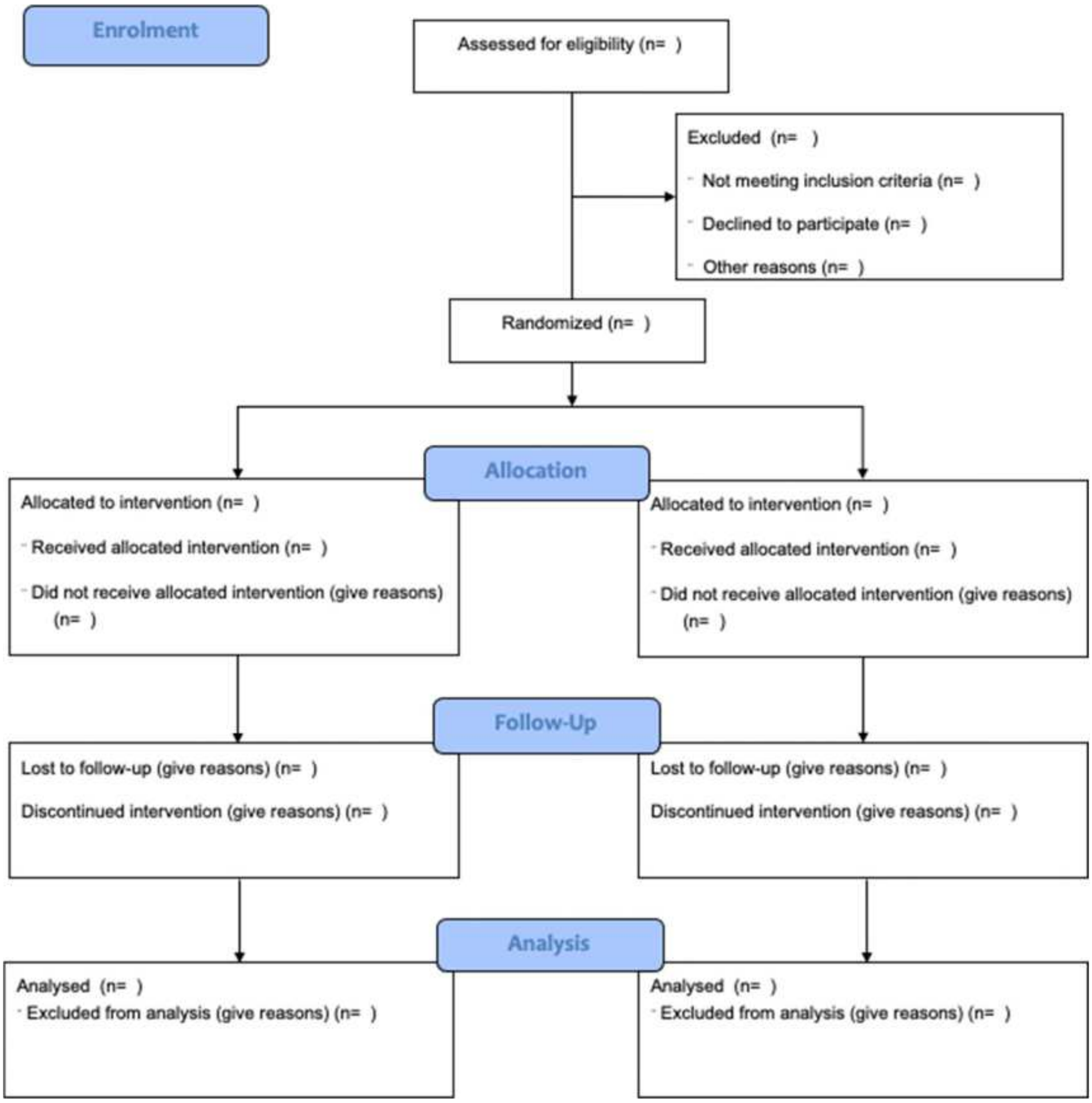

Figure 1: Consort 2010 flow diagram example

5.2 Baseline Characteristics

Baseline characteristics (including medical history and concomitant medications) of participants will be reported by treatment arm. No hypothesis testing will be carried out as any significant differences found are chance-generated (or failure of the implementation of the randomisation) and not for hypothesised reasons.

Continuous variables will be summarised using means and standard deviations (SDs) if (approximately) symmetrically distributed or after appropriately being transformed (e.g. logarithmic) or medians and interquartile ranges (IQRs) if skewed. Categorical variables will be presented as numbers and percentages.

5.3 Outcomes

Descriptive summaries of primary and key exploratory outcomes will be reported based on data from baseline and 52-week follow-up visits and presented in a similar manner as in the descriptive analyses of baseline characteristics.

5.4 Aherence to Treatment and Lost to Follow-up and Pattern of Missingness

The number and proportion of participants, overall and by trial arm, who fail to adhere to treatment over the 52 weeks of the study will be assessed.

The numbers and proportions of participants who are lost to follow-up at each follow-up visit will be summarised in each trial arm. In addition, the pattern/extent of missing data will be quantified using possibly a graphical representation.

6.0 Comparative Analyses

6.1 Analysis of Primary Outcome

For the primary endpoint of annualised change in PET tau standardised uptake value ratio (SUVR), a modified intention to treat (mITT) superiority analysis will be the main analysis for comparing those randomised to the semaglutide treatment arm with those randomised to the placebo arm with respect to their mean annualised change in PET tau SUVR from baseline to 52 weeks in the TS population. An analysis of covariance (ANCOVA)/linear regression, where annualised change in PET tau SUVR is regressed on trial arm after adjusting for baseline PET tau SUVR and the variables used in minimisation (i.e. T2DM status, MCI status and Study Site), will be used to test the primary hypothesis of a treatment effect at the 5%-level of significance. The analysis will be repeated for the completers/complete cases (i.e. those who have both baseline and 52-week PET tau SUVRs).

In addition, a per protocol analysis of compliers will be performed in the PPS population.

Multiple imputation using chained equations[8], under a missing at random (MAR) assumption, will be used to impute missing outcome data. Sensitivity analysis[9] (e.g. tipping-point analysis) to potential informative dropout and low compliance will be considered. Model assumptions (e.g. assumptions of normality and constant variance) will be examined using residual and other diagnostic plots. Estimates of the treatment effect with corresponding standard error (SE) and 95% confidence interval will be reported.

6.2 Analysis of Secondary and Exploratory Outcomes

For continuous key exploratory outcomes measured at baseline and 52 weeks, similar approach to that for the primary analysis will be adopted. That is, an appropriate change measure will be derived and a modified ITT analysis of covariance/linear regression will be used to model the derived change on trial arm, its baseline value and the variables used in minimisation. Both completer and per protocol analyses will be performed. Multiple imputation using chained equations will be used to impute missing data.

For outcomes that are to be measured on more than 2 occasions, methods that take account of the longitudinal nature of the data will be used. Specifically, we will consider using mixed effects (ME) models, which are valid under the MAR assumption as they are likelihood-based. Estimates of the treatment effect with corresponding standard error (SE) and 95% confidence interval will be reported.

6.3 Analysis of Safety Data

Safety data (e.g. adverse events (AEs) and serious adverse events (SAEs)) will be compared between the two trial arms either by comparing the mean rate of events or the proportion of participants experiencing an event in each arms over the follow-up period (including the follow-up call). The estimated risk ratios or absolute risk differences, whichever more appropriate, will be reported with confidence intervals.

7.0 Software

7.1 Data Management

The EDC system used for the trial will be OpenClinica version 4.0. It has been validated by the DTU in accordance with IT005 "Validation of Computerised Systems" as a Good Clinical Practice (GCP) compliant system and is currently operating in a validated state.

The full specification of the database is documented in the Data Specification (DAS), which will be updated to reflect any changes made throughout the study.

Once 20% of participants have completed the trial, a blinded dataset will be made available to the University of Cambridge MRC Biostatistics Unit (MRC BSU) via the DTU secure server to facilitate the construction and testing of analytic programs/analysis scripts. The full dataset will be transferred securely after database lock. Any data quality or management issues that arise after transfer will be resolved between the DTU and the MRC BSU.

7.2 Statistical Analysis

All statistical analyses will be carried out using the R statistical software environment[10] and/or STATA software, version 15[11] or higher. The analyses will be conducted by the MRC BSU. Analyses will be performed blinded to the disclosure of trial arms. An independent statistician who has access to the analysis plan and the full cleaned and quality assured dataset will repeat the primary analysis to ensure reproducibility. Any discrepancies (beyond those due to the effect of random number generation from statistical methods such as multiple imputation) between the findings from the independent statistician and the MRC BSU will be resolved by:

1. Assessing the consistency of their primary analysis plans with those set out in the SAP;
2. Comparing their primary analysis scripts; and
3. Referring to a third party if resolution cannot be achieved between the independent statistician and the MRC BSU.

8.0 References

1. International Conference on Harmonization (ICH). Statistical Principles for Clinical Trials E9.1998. Available at www.ich.org.

2. Schulz KF, Altman DG, Moher D; CONSORT Group. CONSORT 2010 statement: updated guidelines for reporting parallel group randomised trials. BMJ 2010; 340:c332.
3. Jack CR, Knopman DS, Jagust WJ, Petersen RC, Weiner MW, Aisen PS, Shaw LM, Vemuri P, Wiste HJ, Weigand SD, Lesnick TG. Tracking pathophysiological processes in Alzheimer's disease: an updated hypothetical model of dynamic biomarkers. The lancet neurology. 2013;12(2):207-16.
4. Jack CR, Bennett DA, Blennow K, Carrillo MC, Feldman HH, Frisoni GB, Hampel H, Jagust WJ, Johnson KA, Knopman DS, Petersen RC. A/T/N: An unbiased descriptive classification scheme for Alzheimer disease biomarkers. Neurology. 2016;87(5):539-47.
5. ONS https://www.ons.gov.uk/peoplepopulationandcommunity/healthandsocialcare/causesofdeath/datasets/leadingcausesofdeathuk
6. Hanseeuw BJ, Betensky RA, Jacobs HIL, et al. Association of Amyloid and Tau With Cognition in Preclinical Alzheimer Disease: A Longitudinal Study. JAMA Neurol. 2019; 76(8):915–924. doi:10.1001/jamaneurol.2019.1424
7. Whittington, Alex, and Roger Gunn. TauIQ-A canonical image based algorithm to quantify tau PET scans. Journal of Nuclear Medicine. 2021.
8. White IR, Royston P, Wood AM. Multiple imputation using chained equations: issues and guidance for practice. Statistics in medicine. 2011; 30(4):377-99.
9. White IR, Horton NJ, Carpenter J, Pocock SJ. Strategy for intention to treat analysis in randomised trials with missing outcome data. BMJ 2011; 342:d40
10. R Development Core Team (2020). R: A Language and Environment for Statistical Computing. Vienna, Austria: R Foundation for Statistical Computing. ISBN 3-900051-07-0, http://www.R-project.org.
11. StataCorp. 2017. *Stata Statistical Software: Release 15.* College Station, TX: StataCorp LLC)

9.0 Approval

Date:

Current Version:

Version and Date of SAP Amendments:

Signatures

**Trial Role**	**Name**	**Signature**	**Date**
Investigator Statistician	Dr Brian Tom	B Tom<br>23/10/2023 09:40:46	23/10/2023
Chief Investigator	Dr Ivan Koychev	I Koychev<br>23/10/2023 10:27:42	23/10/2023
TSC Chair(s)	Dr Ivan Koychev	I Koychev<br>23/10/2023 10:27:42	23/10/2023

	Professor Rury Holman	R Holman<br>23/10/2023 09:58:41<br>Rury Holman	23/10/2023
DMC Chair	Professor Cornelia van Duijn	C V Duijn<br>23/10/2023 19:36:10<br>Cornelia van Duijn	23/10/2023
DTU Statistician	Dr Ruth Coleman	R Coleman<br>23/10/2023 09:24:35<br>R L Coleman	23/10/2023

10.0 Appendix

10.0 Table of Contents for Output

10.1 Tables

1. Site recruitment by calendar month

Month	Site 1	Site 2	Site 3	Site 4	Site 5	Overall
1st Month						
2nd Month						
3rd Month						
…						
**Total**						

2. Randomisation by arm

	**Arm A**<br>**N (% total)**	**Arm B**<br>**N (% total)**	**Total**
**T2DM**<br>No<br>Yes			
**MCI**<br>No<br>Yes			
**Site**<br>Site 1<br>Site 2<br>Site 3<br>Site 4<br>Site 5			
**Overall**			

3. Adherence/Compliance by arm over 52-week follow-up – cumulative by week

Week	Total no. patients	Arm A<br>N (% total)	Arm B<br>N (% total)	% patients that comply from total
Week 1				
Week 2				
Week 3				
Week 4				
Week 5				
Week 6				
…				
Week 51				
Week 52				
**Total**				

4. Number of subjects attending follow-up assessment visit by arm

**Visit assessment**	**Arm A** N (% - no. subject at visit/total no. of subjects randomised to arm)	**Arm B** N (% - no. subject at visit/total no. of subjects randomised to arm)	**Total no. of subjects attending visit**
4 weeks			
8 weeks			
26 weeks			
39 weeks			
52 weeks			

5. Baseline information by arm

Variables	Arm A	Arm B
Age (yrs)	Mean (SD)	Mean (SD)
Gender		
Female	% (n)	% (n)
Male	% (n)	% (n)
Ethnicity		
White	% (n)	% (n)
Non-white	% (n)	% (n)
Medical history	% (n)	% (n)
Concomitant medication	% (n)	% (n)
Blood pressure	Mean (SD)	Mean (SD)
Heart rate	Mean (SD)	Mean (SD)
Blood markers	Mean (SD)	Mean (SD)
Cognitive test scores	Mean (SD)	Mean (SD)
Depression scores	Mean (SD)	Mean (SD)
Quality of life	Mean (SD)	Mean (SD)
Anxiety Scores	Mean (SD)	Mean (SD)
PET tau SUVR	Mean (SD)	Mean (SD)
PET TSPO SUVR	Mean (SD)	Mean (SD)
etc		

6. Annualized change in outcome by arm

Variables	Arm A	Arm B
PET tau SUVR	Mean (SD)	Mean (SD)
PET TSPO SUVR	Mean (SD)	Mean (SD)
Hippocampal Volume	Mean (SD)	Mean (SD)
CES-D	Mean (SD)	Mean (SD)
HAI	Mean (SD)	Mean (SD)
EQ-5D-5L	Mean (SD)	Mean (SD)
etc		

This Document has been Signed with a **secure electronic signature** via E-Sign.

Envelope Details

**Title**	20231020_ISAP_Statistical Analysis Plan_v1
**Author**	Ada Tse (@ Diabetes Trials Unit) (ada.tse@dtu.ox.ac.uk)
**Envelope Created on**	Mon, 23 Oct 2023 09:11:54
**Envelope ID**	54e08cc4-0d2e-4185-9d84-2287f4ade13f

Document Details

**Title**	20231020_ISAP_Statistical Analysis Plan_v1
**Digital Fingerprint**	7685a8bb-24ad-4eba-a392-602c453b2ab6

Document Signers Scan/Click the QR Code to view signature information

**Name**	Brian Tom
**Email**	brian.tom@mrc-bsu.cam.ac.uk
**Status**	**SIGNED** at Mon, 23 Oct 2023 09:40:46 BST(+0100)
**Signature Fingerprint**	a81f1d65-a221-4836-ac63-0285202bba97

**Name**	Ivan Koychev
**Email**	ivan.koychev@psych.ox.ac.uk
**Status**	**SIGNED** at Mon, 23 Oct 2023 10:27:42 BST(+0100)
**Signature Fingerprint**	38760688-3022-402d-a7b6-2b34efe8d253

**Name**	Rury Holman
**Email**	rury.holman@dtu.ox.ac.uk
**Status**	**SIGNED** at Mon, 23 Oct 2023 09:58:41 BST(+0100)
**Signature Fingerprint**	62d62732-6323-4202-bc5f-167f8cb18a3c

**Name**	Cornelia van Duijn
**Email**	Cornelia.vanDuijn@ndph.ox.ac.uk
**Status**	**SIGNED** at Mon, 23 Oct 2023 19:36:10 BST(+0100)
**Signature Fingerprint**	75c486da-6c45-40f7-a6c8-a5edfc6e6673

ico. Information Commissioner's Office
British Assessment Bureau UKAS MANAGEMENT SYSTEMS 8289 ISO 27001 INFORMATION SECURITY MANAGEMENT
legislation.gov.uk
DEPARTMENT OF COMMERCE • UNITED STATES OF AMERICA •

**Name**	Ruth Coleman
**Email**	ruth.coleman@dtu.ox.ac.uk
**Status**	**SIGNED** at Mon, 23 Oct 2023 09:24:35 BST(+0100)
**Signature Fingerprint**	79afd372-ff5b-4497-a76f-09e85da24b4c

Document History

**Mon, 23 Oct 2023 19:36:10**	**Cornelia van Duijn** Signed the Document (IP: 216.213.156.253)
**Mon, 23 Oct 2023 10:27:43**	**Ivan Koychev** Signed the Document (IP: 192.76.8.64)
**Mon, 23 Oct 2023 09:58:42**	**Rury Holman** Signed the Document (IP: 86.131.153.217)
**Mon, 23 Oct 2023 09:40:47**	**Brian Tom** Signed the Document (IP: 193.60.86.208)
**Mon, 23 Oct 2023 09:24:36**	**Ruth Coleman** Signed the Document (IP: 85.255.237.26)

ico. Information Commissioner's Office
British Assessment Bureau UKAS MANAGEMENT SYSTEMS 8289 ISO 27001 INFORMATION SECURITY MANAGEMENT
legislation.gov.uk
DEPARTMENT OF COMMERCE UNITED STATES OF AMERICA
