# Supplementary material for: Protocol for a double-blind placebo-controlled randomised controlled trial assessing the impact of oral semaglutide in amyloid positivity (ISAP) in community dwelling UK adults
Source: BMJ Open. 2024 Jun 20;14(6):e081401. doi: 10.1136/bmjopen-2023-081401 (PMC11328662; doi:10.1136/bmjopen-2023-081401)
Supplement: Supplementary file 3 [file bmjopen-2023-081401.reviewer_comments.pdf]

## PEER REVIEW HISTORY

BMJ Open publishes all reviews undertaken for accepted manuscripts. Reviewers are asked to complete a checklist review form (**http://bmjopen.bmj.com/site/about/resources/checklist.pdf**) and are provided with free text boxes to elaborate on their assessment. These free text comments are reproduced below.

## ARTICLE DETAILS

| | |
|---|---|
| **TITLE (PROVISIONAL)** | Protocol for a Double-Blind Placebo-Controlled Randomised Controlled Trial Assessing the Impact of Oral Semaglutide in Amyloid Positivity (ISAP) in Community Dwelling UK Adults |
| **AUTHORS** | Koychev, Ivan; Milton, Joanne E.; Butchart, Joe; Hellyer, Peter; Cormack, Francesca; Adler, Amanda; Edison, Paul; Tom, Brian; Hampshire, Adam; Marshall, Charles; Coulthard, Liz; Zetterberg, Henrik; Underwood, Ben; Mummery, Catherine; Holman, Rury |

## VERSION 1 – REVIEW

| | |
|---|---|
| **REVIEWER** | Urich, Thomas<br>University of Southern California, Departments of Medicine |
| **REVIEW RETURNED** | 24-Nov-2023 |

| | |
|---|---|
| **GENERAL COMMENTS** | This is great! I am looking forward to reading more about this study. |

| | |
|---|---|
| **REVIEWER** | Avgerinos, Konstantinos I.<br>Wayne State University |
| **REVIEW RETURNED** | 05-Feb-2024 |

| | |
|---|---|
| **GENERAL COMMENTS** | This is a protocol of a double-blind RCT assessing the effect of the GLP-1 inhibitor semaglutide on tau pathology in individuals aged ≥55 with amyloid positivity. The idea is promising. Overall, I felt that reading through was not smooth and the manuscript has several issues:<br><br>• Beginning with the introduction, the authors mix in the same paragraph the concept of “amyloid positivity” in healthy and the treatments that reduce amyloid accumulation which “have not yielded consistent results.”. However, these treatments are given to individuals with AD and this should be more clear. In the same paragraph, there is an abrupt transition of the need for an “alternative strategy” but it is not stated clearly what this strategy refers to.<br>• The authors need to explain what they mean by “established effects on neural tissue”<br>• “T2D-related confounders”. Please, be more specific.<br>• The authors report that in some studies GLP-1 RA decreased dementia incidence. How is it known that this did not happen through improvement of glycemia and DM, which are both known to increase dementia risk? |

| | |
|---|---|
| | • The authors report that GLP-1 RAs did better compared to other anti-diabetics in reducing dementia. Which other anti-diabetics were tested?<br>• The authors spend several lines to make the argument that tau regulates insulin signaling in neurons. In the trial, the authors test the hypothesis that semaglutide (a drug that may improve glucose transfer through BBB and improves glucose use in the brain) decreased tau pathology, which is the opposite.<br>• The authors state that GLP-1 RA may have an effect on dementia through cerebrovascular and inflammatory factors, but this is vague. Some explanation is needed.<br>• Why semaglutide was chosen out of all GLP-1 inhibitors?<br>• Inclusion and exclusion criteria are missing and should be included<br>• Why age ≥55?<br>• The primary objective paragraph may be confusing the way it's written. Please improve.<br>• "The authors report they will test neuroinflammation by tau PET. How is this possible? Please explain in the text.<br>• I would say cognition rather than "cognitive ability"<br>• "but also more exploratory biomarkers related to inflammation". This is vague<br>• "Otherwise healthy volunteers" may contradict the fact that some had "mild cognitive impairment"<br>• What is "older age" in the stratification algorithm?<br>• The term TSPO is introduced abruptly and naturally that it was never mentioned before in the manuscript. What is TSPO?<br>• What is a "medically trained study investigator"?<br>• How is amyloid positivity determined/quantified?<br>• What is TSPO PET?<br>• Please explain what type of tau pathology is detected by tau PET. Phosphorylated or total or something else?<br>• The duration of the study participation should be apparent early in the protocol<br>• Who is administering cognitive testing?<br>• Why 14 mg dose?<br>• What time will the intervention be administered? With food or without? Any restrictions?<br>• Which studies is the radiation coming from?<br>• How was the selection of T2D individuals at 30% made?<br>• Could you explain what is a mean annual change of 0.05 and 2.01 mean in terms of effect size?<br>• What are the numbers 316, 88, 75? Has the study been completed already?<br>• Do the authors believe the power is adequate or not? This should be mentioned in the text. |

## VERSION 1 – AUTHOR RESPONSE

Reviewer: 1
Mr. Thomas Urich, University of Southern California
Comments to the Author:
This is great! I am looking forward to reading more about this study.
Authors' response: We are grateful for the positive feedback.

Reviewer: 2
Dr. Konstantinos I. Avgerinos, Wayne State University
Comments to the Author:
This is a protocol of a double-blind RCT assessing the effect of the GLP-1 inhibitor semaglutide on tau pathology in individuals aged ≥55 with amyloid positivity. The idea is promising. Overall, I felt that reading through was not smooth and the manuscript has several issues:

• Beginning with the introduction, the authors mix in the same paragraph the concept of "amyloid positivity" in healthy and the treatments that reduce amyloid accumulation which "have not yielded consistent results.". However, these treatments are given to individuals with AD and this should be more clear. In the same paragraph, there is an abrupt transition of the need for an "alternative strategy" but it is not stated clearly what this strategy refers to
Authors' response: We agree that the wording risked conflating the issue of syndromal AD treatment and secondary AD prevention. We have amended our Introduction text to reflect this.

• The authors need to explain what they mean by "established effects on neural tissue"
Authors' response: The references relate to experiments where GLP-1RAs were shown to be neuroprotective in animal models of neurodegeneration. We have amended our text accordingly – Line 117

• "T2D-related confounders". Please, be more specific.
Authors' response: Wium-Andersen study corrected for acute and chronic diabetes complications and adjusted for use of other types of antidiabetic agents. We have added this detail to the text – Line 124
• The authors report that in some studies GLP-1 RA decreased dementia incidence. How is it known that this did not happen through improvement of glycemia and DM, which are both known to increase dementia risk?
Authors' response: The epidemiological studies have accounted for such confounding by controlling for the acute and chronic DM complications – we consider this to be the most reliable proxy of DM control in epidemiological study settings. See original papers we cite for further details.

• The authors report that GLP-1 RAs did better compared to other anti-diabetics in reducing dementia. Which other anti-diabetics were tested?
Authors' response: Wium-Andersen et al. compared against insulin, metformin, sulfonylureas, glitazones, DPP4 inhibitors and SGLT2-inhibitors. Nordgaard, in the nationwide cohort part of the paper, compared against insulin, sulfonylureas, DPP-4 inhibitors and meglitinides. See original papers for further details. Given the limited space in our paper, we feel that a detailed account of these two papers is out of scope and thus we rely on a summary of the findings and referencing.

• The authors spend several lines to make the argument that tau regulates insulin signalling in neurons. In the trial, the authors test the hypothesis that semaglutide (a drug that may improve glucose transfer through BBB and improves glucose use in the brain) decreased tau pathology, which is the opposite.
Authors' response: Respectfully, we do not feel that there is a contradiction. Firstly, the primary outcome of the trial is a reduction in the rate of tau accumulation (rather than a decrease in tau levels). Secondly, we hypothesise that semaglutide interacts with core AD pathology (i.e. tau accumulation). This hypothetical action could take place through neuroinflammatory action,

cerebrovascular or insulin signalling mechanisms; we have thus made an attempt to review the various potential mechanisms.

• The authors state that GLP-1 RA may have an effect on dementia through cerebrovascular and inflammatory factors, but this is vague. Some explanation is needed.
Authors' response: We have added references to the evidence for inflammatory action of GLP-1RAs as requested (Verma et al. 2023; Yoon et al. 2020). We have added the relevant references for reduction in cerebrovascular events (Holman et al. 2017; Rossing et al. 2023).
• The authors state that GLP-1 RA may have an effect on dementia through cerebrovascular and inflammatory factors, but this is vague. Some explanation is needed.
Authors' response: We have amended the sentences in question.

• Why semaglutide was chosen out of all GLP-1 inhibitors?
Authors' response: Public and patient involvement focus groups during the design stages of the project suggested that oral administration would be preferable to subcutaneous administration in this population. Semaglutide was the only oral GLP-1 RA available.

• Inclusion and exclusion criteria are missing and should be included
Authors' response: The detailed inclusion and exclusion are lengthy and so to save space in the paper they are provided in Appendix 1. This has previously been the case for another protocol study published in BMJ Open (https://bmjopen.bmj.com/content/9/3/e024498).

• Why age ≥55?
Authors' response: This age threshold was chosen as previous research from our group has showed that ageing adults begin to accumulate tau rapidly in their mid-50s (https://alz-journals.onlinelibrary.wiley.com/doi/full/10.1002/dad2.12019#:~:text=To%20test%20the%20hypothesis%20that,of%20significant%20increase%20in%20AD).

• The primary objective paragraph may be confusing the way it's written. Please improve.
Authors' response: We have revised the wording – Line 170

• "The authors report they will test neuroinflammation by tau PET. How is this possible? Please explain in the text.
Authors' response: It appears that this comment relates to the original text of the Secondary Analysis subsection (Line 175). We referenced neuroinflammation being investigated using PET and have clarified that we mean TSPO PET.
• I would say cognition rather than "cognitive ability"
Authors' response: We have accepted the change.
• "but also more exploratory biomarkers related to inflammation". This is vague
Authors' response: We have removed the phrase highlighted by the reviewer and instead added a sentence on the end of the paragraph (Lines 187-188) where we specify that plasma will be retained for future exploratory proteomics analyses.
• "Otherwise healthy volunteers" may contradict the fact that some had "mild cognitive impairment"
Authors' response: We accept that the wording can create confusion and have thus removed 'otherwise healthy'. See Line 190 for revised test.

• What is "older age" in the stratification algorithm?
Authors' response: Age is used as a continuous variable in the model; see revised text on Line 197.

• The term TSPO is introduced abruptly and naturally that it was never mentioned before in the manuscript. What is TSPO?
Authors' response: TSPO is now introduced in the first mention of the term in Line 176.
• What is a "medically trained study investigator"?
Authors' response: An investigator with a medical degree and license to practice in the UK. .
• How is amyloid positivity determined/quantified?
Authors' response: Amyloid positivity is determined using PET amyloid or CSF. See Lines 213-222 for details. • What is TSPO PET?
Authors' response: We have now disambiguated the abbreviation in Line 176.
• Please explain what type of tau pathology is detected by tau PET. Phosphorylated or total or something else?
Authors' response: Currently available tau tracers bind to phosphorylated tau.
• The duration of the study participation should be apparent early in the protocol
Authors' response: We have now given the duration of the engagement with the study in the first paragraph of the Methods section (Line 164).
• Who is administering cognitive testing?
Authors' response: These are administered by clinical research staff at each site. As this is standard for clinical trials in the UK, we do not feel that this detail is relevant for the paper.
• Why 14 mg dose?
Authors' response: 14 mg is the standard maintenance dose for semaglutide.
• What time will the intervention be administered? With food or without? Any restrictions?
Authors' response: Standard instructions for the administration of semaglutide are provided to the participants verbally and in written form (information sheet). We have clarified in the text (Lines 276-278): 'Participants to take the drug with half a glass of water and i) to not split, crush or chew the tablet, ii) to take it in the morning before any oral intake, iii) not to eat, drink or take any other medication for 30 minutes after administration.' • Which studies is the radiation coming from?
Authors' response: The radiation exposure calculations are based on National Radiological Protection Board papers as indicated in the text.
• How was the selection of T2D individuals at 30% made?
Authors' response: The capping of the study population to a maximum of 30% T2DM was based on minimise the likelihood that any impact of semaglutide on AD is through a diabetes-specific mechanism.
• Could you explain what is a mean annual change of 0.05 and 2.01 mean in terms of effect size?
Authors' response: The mean annual tau PET changes stem from the two available studies in amyloid positive individuals. These values together with the SD allowed us to generate a range of effect sizes/differences assuming different scenarios of difference between semaglutide and placebo as well as alpha levels. For example, an effect difference/size of 0.01 (placebo vs semaglutide) corresponds to a 20% reduction in the mean annual change of 0.05 when untreated in Table 1. We have now included the effect differences in the Table for clarity.
• What are the numbers 316, 88, 75? Has the study been completed already?
Authors' response: The study is currently recruiting. We calculated that we need to screen 316 individuals to randomise 88 participants which will lead to 75 participants completing the study assuming a 14% drop out rate. We have updated the table legend (Lines 318-319)
• Do the authors believe the power is adequate or not? This should be mentioned in the text.
Authors' response: There are currently no studies examining the effects of semaglutide on tau PET accumulation in preclinical AD. It is our view that the sample size will provide robust estimates of the size of the effect of semaglutide on tau accumulation to guide future confirmatory studies. We have amended lines 316-317 accordingly.
